# The Prevalence of Xerostomia in Older Thai Individuals with Type II Diabetes Mellitus and Its Association with Type of Toothpaste and Oral Functions: A Cross-Sectional Study Using Questionnaires

**DOI:** 10.3390/geriatrics8040076

**Published:** 2023-07-15

**Authors:** Panitan Sonpanao, Kajohnkiart Janebodin, Niwatchai Namvichaisirikul, Supattarayan Thongjit, Paiboon Jitprasertwong

**Affiliations:** 1 Translational Medicine (International Program), Institute of Medicine, Suranaree University of Technology (SUT), Nakhon Ratchasima 30000, Thailand; panitanpage@gmail.com; 2 School of Geriatric Oral Health, Institute of Dentistry, Suranaree University of Technology (SUT), Nakhon Ratchasima 30000, Thailand; 3 Department of Anatomy, Faculty of Dentistry, Mahidol University, Bangkok 10400, Thailand; dtjanebk@gmail.com; 4 Department of Family Medicine and Community Medicine, Institute of Medicine, Suranaree University of Technology (SUT), Nakhon Ratchasima 30000, Thailand; niwatchai@sut.ac.th; 5 School of Family and Community Oral Health, Institute of Dentistry, Suranaree University of Technology (SUT), Nakhon Ratchasima 30000, Thailand; thongjit.sup@g.sut.ac.th

**Keywords:** xerostomia, diabetes, older people, toothpaste, dental health surveys

## Abstract

Aim: To investigate the prevalence of xerostomia in older people with diabetes mellitus and its impacts on oral functions, as well as to determine potential risk factors for xerostomia. Methods: An analytical cross-sectional study was conducted on 623 older type 2 diabetes mellitus (T2DM) Thai people using valid structural questionnaires. Patients were interviewed, and data were recorded. Xerostomia was assessed using subjective symptom questionnaires. Risk factors for xerostomia were analyzed using bivariate and multiple logistic regression analyses. Results: Among the study participants, 38.4% of the older T2DM people had xerostomia, which is associated with sex, age, type of toothpaste, years of diabetes, hemoglobin A1c level, other systemic diseases, medication, smoking, alcohol consumption, and denture wearing. It was significant that xerostomia was associated with toothpaste containing spicy herbal extracts (OR: 9.32 [3.46 to 15.25]), while toothpaste containing artificial sweeteners tended to lower the risk of xerostomia. In addition, older T2DM adults with xerostomia had greater impaired oral functions, which include difficulties in speaking (OR: 3.31 [1.11 to 9.80]), tasting (OR: 5.12 [3.26 to 8.06]), swallowing (OR: 3.59 [2.32 to 5.53]), and chewing (OR: 3.34 [1.15 to 5.82]). Conclusions: Xerostomia is prevalent in older Thai people with T2DM. The results suggest that toothpaste containing spicy herbal extracts might increase the risk of xerostomia, resulting in various oral function problems. Therefore, greater awareness of xerostomia in this group should be raised to monitor dental health, and professionals should work in parallel with other aspects of oral health promotion.

## 1. Introduction

Saliva is a vital component that supports the complete function of the oral tissues. The qualitative and quantitative relationship between salivary secretion and oral pathology leads to hyposalivation and xerostomia. Xerostomia is usually defined as the subjective complaint of a dry mouth [1]. The prevalence of xerostomia is higher in older people, but xerostomia is also common in the normal population and increases with age [2], resulting in discomfort, speaking difficulties, swallowing issues, and impaired taste reception [3]. Older people often experience decreased saliva secretion, causing xerostomia. Xerostomia can cause oral tissue to rupture, which becomes prone to infection, resulting in the deterioration of oral health. Abnormalities of the salivary glands occur as a result of systemic diseases, side effects of radiotherapy and chemotherapy in head and neck cancer, side effects from medications, aging, and diabetes mellitus (DM) [4].

Diabetes can directly or indirectly affect the function and structure of the salivary glands, resulting in insufficient saliva secretion. In 2021, the global prevalence of diabetes was 10.5%, and type II diabetes mellitus (T2DM) accounts for about 90% of all diabetes cases [5]. Therefore, diabetes is a major public health problem worldwide, especially in the older population. According to the IDF (International Diabetes Federation) 2021 survey, the Thai population has a high rate of diabetes, at 11.6% of the population. 

A previous study on the prevalence of xerostomia among older people in Japan reported that 27.3% complained of xerostomia [6]. Another study in Spain has discovered 30.7% [7]. Nevertheless, there are few studies on the prevalence of xerostomia in Thailand, and no previous research has been conducted using prevalence surveys to determine the factors associated with xerostomia. In addition to individual factors, diabetes, and health behaviors, one of our interests and most likely directly related to xerostomia is toothpaste, as it is a basic product for regular and continuous use. This is because diabetics often need to use toothpaste along with their daily brushing. Toothpastes containing different ingredients, specifically toothpastes containing sodium lauryl sulfate (SLS), spicy herbal extracts, and artificial sweeteners, were speculated to be related to the issue of xerostomia. Therefore, various conditions relating to xerostomia should be investigated since there are no existing relevant studies in such a large study population. Moreover, the older people with T2DM in this study are currently the largest group studied in Thailand, so the findings are expected to be useful to improve the quality of life of older people suffering from xerostomia. We hypothesized that the prevalence of xerostomia in older Thai individuals with T2DM is associated with the type of toothpaste and has a direct impact on oral functions. This study, therefore, aimed to investigate the prevalence of xerostomia in older people with DM and its impacts on oral functions, as well as determine potential risk factors for xerostomia.

## 2. Materials and Methods

### 2.1. Study Population and Inclusion Criteria

**Figure 1:**
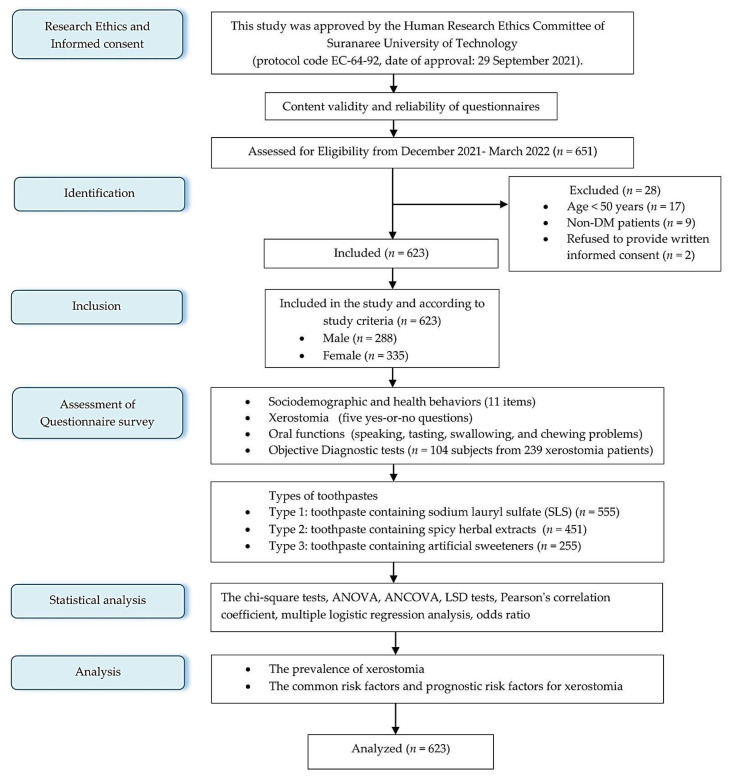
Flow chart diagram for study participants. (*n* = number of participants).

This analytical cross-sectional study was conducted in accordance with the Declaration of Helsinki and approved by the Human Research Ethics Committee of Suranaree University of Technology (protocol code EC-64-92, date of approval: 29 September 2021). Informed consent was obtained prior to inclusion in the study. A total of 651 older individuals who received ongoing care at 2 non-communicable disease clinics (NCDs) of Suranaree University Hospital, Thailand, were registered during the study period. Twenty-eight individuals were excluded, as shown in Figure 1. A total of 623 older T2DM individuals were included in this study. The sample size was calculated using the formula *n* = Z^2^ × *P* (1 − *P*)/d^2^, where *n* = number of T2DM older people, Z = 1.96 (standard normal variance at 95% CI), d = 5% (absolute error or precision), and *P* (expected prevalence) = 27.3% [6]. Thus, the calculated sample size was 305 older subjects, but we collectively surveyed all the patients (*n* = 623) over the course of four successive months (December 2021–March 2022). The inclusion criteria were those who had a diagnosis of T2DM by a medical doctor and were aged 50 years or older. Patients who refused to complete the consent form or who were diagnosed with a mental disorder were excluded from the present study.

### 2.2. Assessment of Xerostomia and Questionnaire Survey

The study was conducted through a questionnaire completed by T2DM older people through face-to-face interviews by the research team in Thai. The content validity of the research instrument was approved by three expert dentists. Content validity was analyzed using the Index of Item Objective Congruence (IOC), and all items were greater than 0.5. The internal consistency (Cronbach’s alpha) of the questionnaire = 0.772, and interpersonal reliability = 0.832. The questionnaire was divided into three sections. The first section (I) collected sociodemographic and health behaviors, separated into 11 items consisting of sex, age, education, years since diabetes diagnosis, hemoglobin A1c (HbA1c) level, systemic disease, medication, smoking, alcohol consumption, toothpaste brands, and denture wearing.

We divided types of toothpastes used by participants into three groups according to the specific ingredients: toothpaste containing sodium lauryl sulfate (SLS) (type 1), toothpaste containing spicy herbal extracts, e.g., *Eugenia Caryophyllus* (clove) leaf oil, clove oil, peppermint oil, spearmint oil, olive oil, menthol, eucalyptus oil, fennel extract, glycyrrhiza extract, cinnamon bark extract, camphor (type 2), and toothpaste containing artificial sweeteners, e.g., sorbitol, xylitol, stevia, aspartame, sodium saccharin, sodium cyclamate, acesulfame potassium, and sucralose (type 3). The fluoride content of toothpastes in this study ranged between 1000 and 1500 ppm (parts per million).

The second section (II) gathered data related to the assessment of xerostomia. Xerostomia was diagnosed according to five yes-or-no questions (Table 1). If a patient answered “Yes” to only 1 question, it was considered xerostomia. These questionnaires were modified from de Carvalho et al. [8]. The third section (III) investigated various oral functions, which consisted of four parts, including speaking, tasting, swallowing, and chewing problems (5-point scale). Swallowing problems were recorded using the 7-point Functional Oral Intake Scale (FOIS), modified from Crary et al. [9], where level 1 denotes nothing by mouth and level 7 denotes total oral intake with no restrictions. Chewing problems were evaluated by asking whether the patients were able to chew the 14 listed food items, which were modified from Kunon and Kaewplung [10]. The 14 Thai foods ranging from easy to difficult to chew were rice soup or porridge; Chinese vegetable stew; clear soup or steamed vegetables; cooked rice; noodles; omelet; steamed fish; sour curry; banana; fried fish; orange; guava; fried pork; and stir-fried vegetables. Chewing ability was scored from 0 to 2, where 0 denotes being unable to chew at all, 1 denotes that it is difficult to chew, and 2 denotes being able to chew well. To interpret the level of speaking, tasting, and swallowing problems, scores were calculated using an assigned mean (good, moderate, and poor levels; for speaking and tasting problems, 1.0–5.0 and 1.0–7.0 in swallowing), and chewing ability used scores 0–28.

### 2.3. Measurement of Salivary Flow Rates and Oral Dryness Examinations

To determine the objective diagnostic tests for hyposalivation status, 104 subjects from a total of 239 xerostomia subjects were randomly tested for salivary flow rate and oral dryness examinations. The unstimulated salivary flow rate was measured using the spitting method [11]. Briefly, the subjects sat comfortably in an upright position. The unstimulated whole saliva was collected between 7.30 and 11.00 a.m. by spitting into a disposable plastic tube. To avoid blood contamination, subjects were instructed to spit gently every 30 s for 5 min. The volumes of saliva samples were recorded and expressed in milliliters per minute.

In addition, the modified cotton method [12] was performed on the same subjects by placing cotton under and over the tongue for 30 s and measuring the weight of the saliva absorbed by the cotton. A diagnosis of hyposalivation is considered whole unstimulated saliva < 0.1 mL/min [13], and oral moisture < 0.02 g of saliva collected at the surface of the tongue or <0.1 g at the hypoglossus is considered oral dryness [12].

### 2.4. Statistical Analysis

Statistical analyses were performed with SPSS version 25 (IBM Corp., Armonk, NY, USA). The chi-square tests, analysis of variance (ANOVA), analysis of covariance (ANCOVA), and least significant difference (LSD) tests were used to compare the difference in frequency distribution and mean. Continuous variables are described as means and standard deviations (m ± SD). Relationships between variables were investigated using chi-square and Pearson’s correlation coefficient, while bivariate regression and multiple logistic regression analysis were used for predicting and computing adjusted odds ratios with 95% confidence intervals and were set at a two-sided *p*-value < 0.05, which was accepted as the level for statistical significance.

## 3. Results

### 3.1. Prevalence of Xerostomia

The summary of study participants is shown in the study flow chart diagram (Figure 1).

The screening of xerostomia in a total of 623 T2DM patients was based on subjective symptom questionnaires. We found that 239 patients (38.4%) reported at least 1 symptom and were considered xerostomia patients. Accordingly, the objective diagnostic tests were randomly assigned to 104 subjects from 239 xerostomia patients to explore hyposalivation status. The subjective symptom scores were calculated, and the results of objective diagnostic tests are shown in Table 1 (*n* = 104). The mean subjective symptom scores in xerostomia subjects were 2.27 ± 1.09. The unstimulated saliva in xerostomia subjects was 0.085 ± 0.012 mL/min. The amount of oral moisture at the surface of the tongue was 0.01 ± 0.005 g and was 0.09 ± 0.03 g at the hypoglossus. The xerostomia patients’ salivary flow rates and oral moisture tests were considered hyposalivation and oral dryness.

The majority of the patients complained of at least one symptom, which was too little saliva in their mouth (33.7%). The overall prevalence of xerostomia was 38.4%, of which 43.3% were female and 32.6% were older males (Table 2). The xerostomia prevalence was significantly higher in participants over 59 years old than in 50–59 year olds (27.3%).

### 3.2. Xerostomia in Relation to Diabetic Status and Toothpaste

The prevalence was comparatively higher in older people who routinely used toothpaste containing spicy herbal extracts (39.4%), whereas patients who used toothpaste containing artificial sweeteners tended to have a lower prevalence of xerostomia (36.4%), as shown in Table 2. Nevertheless, none of the diabetic patients who used hydrating toothpaste were reported.

The prevalence of xerostomia in older individuals categorized by diabetes profiles is shown in Table 2. The prevalence of xerostomia is significantly associated with years since diabetes diagnosis, as the highest occurrence of xerostomia (59.1%) was reported in individuals who were diagnosed with T2DM more than ten years ago. In fact, the lowest occurrence (16.3%) was observed in a patient who had been diagnosed with diabetes for less than 6 years. We found that the occurrence of xerostomia was not dose-dependent on HbA1c levels. However, a relatively high prevalence of xerostomia was noted in patients who presented with other systemic diseases such as cardiovascular disorders (88.8%), dyslipidemia (50.1%), and hypertension (50.1%). Furthermore, a fairly high prevalence (60.0% and 63.6%, respectively) was also observed in patients who were under-prescribed antihypertensive drugs and cardiovascular medications (Table 2).

In the present study, only 42 patients (6.7%) were reported to be wearing dentures. The highest occurrence of xerostomia (100%) was observed in patients who had complete dentures, whereas a lower prevalence was found in those who had removable partial dentures (86.9%) and fixed partial dentures (23.0%).

### 3.3. The Prevalence of Xerostomia Related to Oral Function Problems

As shown in Table 3, most of the participants reported issues with swallowing (59.4%), while smaller proportions reported issues with chewing (0.6%), speaking (3.5%), and tasting (26.6%). Most of the participants considered themselves to have good chewing abilities (99.4%). The foods they complained about being difficult to chew or not possible to chew were fried pork (67.5%) and guava (33.3%).

The data from Table 3 showed various significant factors causing the four oral function problems. Described as speaking problems, we found that the important variables were complete denture wearing and xerostomia. Tasting problems were related to two factors, including over 5 years since diabetes diagnosis and xerostomia. Swallowing problems were associated with removable partial denture wearing and xerostomia. The most common causes of chewing problems were denture wearing (complete dentures) and xerostomia.

### 3.4. Factors Associated with Xerostomia

In the bivariable analysis (Table 4), 11 of 17 variables were significantly associated with the occurrence of xerostomia. Of these, eight were sociodemographic factors, and three were related to diabetes factors (years since diabetes diagnosis, having systemic diseases other than diabetes, and having medications) and daily toothpaste used by older people. The results showed that those factors were significantly associated with the occurrence of xerostomia (*p* < 0.001), which included over ten years since diabetes diagnosis, HbA1c ≥ 7%, having systemic diseases other than diabetes, taking medications, smoking, alcohol consumption, and denture wearing. Education and SLS-free toothpaste were not shown to be associated with xerostomia. The details are shown in Table 4, and there is no multicollinearity.

The results of the analysis revealed that the factors relating to significant xerostomia included female, aged over 59, over 5 years since diabetes diagnosis, HbA1c ≥ 7%, other systemic diseases, medications, smoking, alcohol consumption, denture wearing, and toothpaste containing spicy herbal extracts.

On the contrary, we discovered that toothpaste containing artificial sweeteners tended to reduce the risk of xerostomia (OR = 0.87, 95% CI = 0.62 to 1.21), and toothpaste containing sodium lauryl sulfate was not associated with xerostomia (OR = 1.00, 95% CI = 0.59 to 1.68). It is interesting that the type of toothpaste affects the occurrence of xerostomia in both well-controlled (HbA1c ≤ 6.5%) and uncontrolled T2DM patients (HbA1c > 6.5%). Significantly, the prevalence of xerostomia in uncontrolled T2DM patients using toothpaste containing spicy ingredients was found to be 4.34 times higher than was the case for well-controlled patients (Table 4).

Multivariate logistic regression analysis (Table 5) showed prognostic risk factors for xerostomia (in multiple logistic regression, with a *p*-value < 0.05 in the bivariable analysis or in combination with 95% confidence interval consideration). After adjusting for potential confounding factors (systemic diseases other than diabetes, medications, smoking, and alcohol consumption). Of these, eight variables were identified as significant prognostic risk factors (Table 5). Prognostic risk factors were female (OR = 1.36, 95% CI = 1.21 to 2.61), age 60–69 years (OR = 1.51, 95% CI = 1.29 to 1.89), age > 69 years (OR = 2.31, 95% CI = 1.84 to 2.53), toothpaste containing spicy herbal extracts (OR = 9.32, 95% CI = 3.46 to 15.25), more than 10 years since diabetes diagnosis (OR = 2.40, 95% CI = 0.23 to 4.68), HbA1c ≥ 7% (OR = 8.17, 95% CI = 2.08 to 12.34), and removable partial denture wearing (OR = 8.59, 95% CI = 1.60 to 12.55). However, we found that toothpaste containing artificial sweeteners was a protective factor for xerostomia (OR = 0.35, 95% CI = 0.02 to 4.82).

## 4. Discussion

### 4.1. Xerostomia

Dry mouth was common in the general population, with higher rates observed among older people [14]. Significantly, the prevalence of xerostomia is increased in diabetic patients [15]. For these reasons, the prevalence of xerostomia in our studied population was 38.4%, which is greater than in previous research that was conducted in Asia and Europe (27.3% and 30.7%, respectively) [6,7]. Moreover, the present study demonstrated that xerostomia is more common in females (43.3%), which is similar to the findings of Shirzaiy and Bagheri [16]. Consistent with other studies [17], menopause and hormonal changes are seen as potential xerostomia risk factors, and menopausal women frequently complain of oral dryness. Additionally, they discovered that menopausal women had significantly lower mean unstimulated salivary flow rates than male controls. The hormonal changes associated with menopause in women may increase the risk of developing xerostomia.

### 4.2. Type of Toothpaste

Nearly 90% of DM older people use toothpaste that contains SLS. This study revealed that the majority of toothpaste ingredients utilized SLS synthetic compounds (9 out of 13 toothpaste brands in this study). SLS was contained in the majority of the toothpaste ingredients [18]. There were patients who chose toothpaste containing spicy herbal extracts (451 cases), probably because of Thai food culture or because most Thai people have a preference for spicy food [19]. This study’s investigation of toothpaste revealed that the majority of toothpaste available on the Thai market frequently contains both hot and cold spice ingredients. Consistent with the results of this study, 72.4% of diabetic patients chose toothpaste containing spicy herbal extracts that may irritate oral tissues, causing mucosal desquamation.

In this study, we found that each brand of toothpaste used by diabetic patients contained artificial sweeteners such as sodium saccharin, sorbitol, and xylitol, and 41.0% of these 623 cases used toothpaste containing artificial sweeteners. This is consistent with the current toothpaste industry, which often adds artificial sweeteners to flavors for the purpose of breath-freshening users [20]. In addition, sugar-free and artificially sweetened products were found in various supermarkets as well [21].

### 4.3. Oral Function Problems

More than half of the population had problems swallowing. The results were consistent with the chewing problems. Patients complained that the most difficult foods to chew were fried pork, guava, and fried fish, which are relatively dry and hard foods. These problems can make it difficult to consume food, resulting in a lack of good nutrients for their health. In the long term, this will increase the chance of developing a health problem. Therefore, the type of food should be considered an important variable for these patients.

### 4.4. The Association between Diabetes, Xerostomia, Type of Toothpaste, and Oral Function Problems

The results of the present study also demonstrated that higher HbA1c levels and a longer duration of diabetes were associated with xerostomia. Recently, studies have shown that xerostomia and hyposalivation may not always be concomitant [22]. The diagnosis of xerostomia requires a thorough history of the patient’s reported symptoms of oral dryness, medication use, and past medical history [23]. Several questionnaires have been proposed to identify patients with xerostomia. However, it was found to have high sensitivity but low specificity for hyposalivation. Interestingly, xerostomia patients frequently do not show any objective signs of hyposalivation. A diagnosis of hyposalivation is made when the stimulated salivary flow rate is ≤ 0.5–0.7 mL/min and the unstimulated salivary flow rate is ≤ 0.1 mL/min. Patients’ subjective symptoms or self-reported feeling of dry mouth alone are not always parallel to the real signs of xerostomia and/or hyposalivation [13]. Therefore, the diagnosis of xerostomia and true hyposalivation is dependent upon a careful and detailed history and objective diagnostic tests such as salivary flow rates and oral moisture tests. It is noted that in the present study, patients were screened for xerostomia solely due to subjective symptoms of xerostomia, and we discovered that the longer diabetes has existed, the higher the prevalence of xerostomia, especially in women. In fact, the results of objective diagnostic tests confirmed that xerostomia scores show a positive relationship with objective diagnostic tests, including salivary flow and oral moisture tests (surface of the tongue/hypoglossus). The findings were consistent with several previous studies [24,25,26,27,28,29,30], which reported that there was a statistically significant positive correlation between hyposalivation and subjective symptoms of xerostomia. However, to confirm that all patients have true hyposalivation, the measurement of salivary flow rate in combination with the xerostomia questionnaire should be performed for all subjects in a further modest study.

Hyperglycemia in diabetes usually causes functional damage to many organ systems. In addition, mitochondrial dysfunction can be generated. Mitochondria play an important role in regulating salivary secretion. Once it functions improperly, salivary gland dysfunction can occur in diabetic patients [31]. In addition, neuropathic and microvascular abnormalities, including endothelial dysfunction and deterioration of microcirculation, that are associated with DM may play a role in disturbed salivary flow and composition. Persistent hyperglycemia caused by diabetes dysregulation may result in water loss, but diabetes-related structural changes in the salivary glands and dehydration associated with elevated blood glucose may increase osmotic gradients within the salivary glands [32]. This was related to alterations in the microcirculation to the salivary glands, destruction of the gland parenchyma, dehydration, and complications with glycemic control [33], consistent with findings from previous studies [34,35,36,37], which reported evidence of the deregulation of specific salivary proteins such as salivary amylase levels, aquaporins, nitric oxide synthase, and tetrahydrobiopterin protein (NOS-BH4), a statherin protein relevant to alterations in salivary gland morphology, cellular architecture, and general salivary secretion and composition associated with diabetes development. Additionally, there is a tendency for the association between hyposalivation and poor metabolic control of DM to progress in the same direction. According to previous studies, the reduction in salivary flow was directly associated with DM patients’ poor metabolic control, while hyposalivation and xerostomia have been related to high HbA1c and fasting plasma glucose due to DM, which affects the cellular microenvironment in many different kinds of organ systems, including the kidneys, blood vessels, nerves, eyes, and nervous system. No exception is the oral cavity; diabetes has a significant impact on oral tissues, especially for individuals who suffer from poor glycemic control [38]. Furthermore, people with poorly controlled DM tended to show a significantly lower response to the parotid glandule’s stimulation of salivary secretion. They suggest that diabetics may have impaired salivary flow in comparison to non-diabetic individuals [39] and that DM patients with poor glycemic control have considerably decreased salivary flow rates [40].

A previous study [41] suggested that a medical condition other than diabetes should be of concern in older patients. For instance, polymedications such as anticholinergics and antidepressant drugs may result in xerostomia. These medications were shown to be significantly linked to xerostomia caused by their xerogenic effects.

The study found that smoking, alcohol consumption, and denture wearing were also responsible for xerostomia since salivary macromolecules, enzymes, and proteins that serve as defense mechanisms are destroyed by tobacco smoke. The salivary glands are adversely affected by smoking tobacco, which also decreases the quality and flow of the saliva [42]. Furthermore, alcohol consumption has a diuretic impact, which can cause dehydration and enhance xerostomia [43]. In addition, [44] reported that denture wearing could cause xerostomia due to the possibility of denture stomatitis from the increased dental tissue–denture contact. Moreover, wearing ill-fitting dentures may result in decreased saliva secretion.

We found that 39.4% of diabetic patients who used toothpaste containing spicy herbal extracts had xerostomia, and we also found that in these types of toothpaste, there were spicy or cold ingredients such as menthol, clove, etc. Long-term usage of this type of toothpaste may increase the risk of xerostomia (Table 2, Table 4 and Table 5) since it is often found that people with xerostomia already have altered tastes or intolerances to spicy or sour tastes [45].

On the other hand, in this study’s research, we discovered that the risk of xerostomia among individuals with diabetes was decreased by toothpaste containing artificial sweeteners. In a similar previous study, it was discovered that artificial sweeteners such as xylitol can improve salivary flow rates in patients receiving intensity-modulated radiation therapy for head and neck cancers without causing significant side effects [46] and were effective in alleviating the manifestations of dry mouth [47]. Additionally, it was reported that products containing olive oil, betaine, and xylitol that were structured like toothpaste, gel, and spray considerably reduced the majority of symptoms and the constraints on quality of life caused by dry mouth in patients receiving radiation. In particular, xylitol plays an important role in salivary stimulation activity [48]. In the present study, we showed that the types of toothpastes affect the prevalence of xerostomia in T2DM patients, whether they have controlled or uncontrolled DM. Specifically, uncontrolled T2DM patients using toothpaste containing spicy extracts are more likely to have xerostomia than those with well-controlled T2DM. This finding suggests that using toothpaste containing spicy extracts may contribute to the development of xerostomia in poorly controlled T2DM (Table 4).

The results confirm that xerostomia causes problems with all aspects of oral function (Table 3) and other oral diseases due to saliva serving a variety of crucial functions. We also observed that the association between sex and the number of years since developing diabetes with xerostomia is intriguing. Because the numbers were comparable in each group (Table 2), this could have had an impact on xerostomia. Notwithstanding, we discovered in the present study that the type of toothpaste was related to xerostomia (Table 5). In order to reduce the symptoms of xerostomia, choosing the appropriate toothpaste for diabetic patients is a consideration that should not be disregarded. Although smoking, drinking alcohol, and denture wearing are all associated with xerostomia, the number of patients in each category varies greatly. Further studies should be focused on a smaller proportion of the population and involve the study of healthy older individuals or other patient groups to investigate xerostomia trends and their prevalence in diverse populations. The limitations of the present study were that we did not exclude diabetic patients with other diseases and included them in calculating the prevalence of xerostomia because we wanted the study population to be broadly representative of the general population to increase the generalizability of the findings. Additionally, a further study should be conducted to investigate objective diagnostic tests such as salivary flow rate combined with the xerostomia questionnaire to reflect the functional and physical status of the salivary glands. Furthermore, data on the fluoride content of toothpaste and the length of time patients brush their teeth should be considered because these issues might contribute to xerostomia.

The findings in the present study indicate that older people with DM are more prone to xerostomia. Dental health personnel should monitor and emphasize that patients are aware of the risk behaviors that can result in this problem by consulting a doctor for appropriate care to help reduce or alleviate the severity of xerostomia to the greatest feasible extent.

## 5. Conclusions

The prevalence of xerostomia in older Thai individuals with T2DM in this study is 38.4%. The key risk factors are using toothpaste containing spicy ingredients, denture wearing, poor glycemic control, and the duration of diabetes, respectively. The presence of xerostomia has a direct and negative impact on oral functions.

## Figures and Tables

**Table 1:** Xerostomia assessment in the studied population and objective diagnostic tests of xerostomia cases.

Subjective Evaluation of Xerostomia *n* = Number of Participants	Yes	No
	*n* (%)	*n* (%)
1. Do you feel you have too little saliva in your mouth?	210 (33.7)	413 (66.3)
2. Do you have a dry mouth when you eat meals?	63 (10.1)	560 (89.9)
3. Do you often have a dry mouth at night or when you wake up in the morning?	172 (27.6)	451 (72.4)
4. Do you feel that swallowing your food is difficult?	58 (9.3)	565 (90.7)
5. Do you sip water all the time while swallowing food?	30 (4.8)	593 (95.2)
**Total (623)**	**239 (38.4)**	**384 (61.6)**
Subjective symptom scores and objective diagnostic tests (*n* = 104 cases from 239 xerostomia patients)	Mean	SD
Xerostomia screening	2.27	1.09
Salivary flow rate measurement (mL/min)	0.085	0.012
Oral moisture (surface of the tongue) (g)	0.01	0.005
Oral moisture (hypoglossus) (g)	0.09	0.03

**Table 2:** Prevalence of xerostomia and differences in sociodemographic data and health behaviors of 623 older patients with T2DM.

Variables	Categories	*n* (%)	No. of Xerostomia Cases (%)	*p*-Value
Sex	Male	288 (46.2)	94 (32.6)	0.006 *
	Female	335 (53.8)	145 (43.3)	
Age (years)	50–59	143 (23.0)	39 (27.3)	0.002 *
	60–69	320 (51.3)	141 (44.1)	
	Over 69	160 (25.7)	59 (36.8)	
	Mean = 65.48, SD = 7.73, min. = 50, max. = 88			
Toothpaste (type 1)	SLS-free	68 (11.0)	26 (38.2)	0.982
	Containing SLS	555 (89.0)	213 (38.3)	
Toothpaste (type 2)	Spicy herbal extracts-free	172 (27.6)	61 (35.4)	0.358
	Containing spicy herbal extracts	451 (72.4)	178 (39.4)	
Toothpaste (type 3)	Artificial sweeteners-free	368 (59.0)	146 (39.6)	0.419
	Containing artificial sweeteners	255 (41.0)	93 (36.4)	
Education	None	12 (1.9)	4 (33.3)	0.468
	Elementary school	148 (23.8)	52 (35.1)	
	High school	282 (45.3)	115 (40.7)	
	Bachelor’s degree	162 (26.0)	58 (35.8)	
	Higher than bachelor’s degree	19 (3.0)	10 (52.6)	
Years since diabetes diagnosis	0–5	147 (23.6)	24 (16.3)	<0.001 **
	6–10	241 (38.7)	76 (31.5)	
	Over 10	235 (37.7)	139 (59.1)	
	Mean = 10.13, SD = 5.57, min. = 0.10, max. = 32.00			
HbA1c (%)	≤6.5	134 (21.5)	23 (59.1)	<0.001 **
	6.6–6.9	126 (20.2)	32 (25.3)	
	≥7	363 (58.3)	184 (50.6)	
	Mean = 8.38, SD = 1.97, min. = 5.70, max. = 13.20			
Systemic diseases other than diabetes	None	135 (21.7)	16 (11.8)	<0.001 **
	Hypertension	411 (66.0)	206 (50.1)	<0.001 **
	Dyslipidemia	381 (61.2)	191 (50.1)	<0.001 **
	Cardiovascular disorders	9 (1.4)	8 (88.8)	0.002 *
	Thyroid disorders	21 (3.4)	13 (61.9)	0.024 *
	Hematologic disorders	11 (1.8)	8 (72.7)	0.018 *
	Renal disorders	67 (10.8)	40 (59.7)	<0.001 **
	Respiratory disorders	55 (8.8)	25 (45.4)	0.257
	Allergy	109 (17.5)	47 (43.1)	0.261
	Gout	42 (6.7)	28 (66.6)	<0.001 **
Medications	None	137 (22.0)	22 (16.0)	<0.001 **
	Antihypertensive medication	404 (64.8)	206 (60.0)	<0.001 **
	Antidyslipidemic agents	359 (57.6)	186 (51.8)	<0.001 **
	Antiplatlet and anticoagulant medication	346 (55.5)	163 (47.1)	<0.001 **
	Pain medication	199 (31.9)	77 (38.6)	0.907
	Gastrointestinal agents	16 (2.6)	7 (43.7)	0.653
	Cardiovascular medication	11 (1.8)	7 (63.6)	0.082
	Antihistamine	79 (12.7)	30 (37.9)	0.939
Smoking (cigarettes per day)	Never	602 (96.6)	220 (36.5)	<0.001 **
	1–5	21(3.4)	19 (90.4)	
	Mean = 0.09, SD = 0.54, min. = 0, max. = 5			
Alcohol consumption frequency	Never	535 (85.9)	172 (32.1)	<0.001 **
	Monthly or less	41 (6.6)	31 (75.6)	
	2–4 times a month	31 (5.0)	22 (70.9)	
	2–3 times a week	11 (1.7)	10 (90.9)	
	4 or more times a week	5 (0.8)	4 (80.0)	
Denture wearing	None	581 (93.3)	210 (36.1)	<0.001 **
	Complete dentures	6 (0.9)	6 (100)	
	Removable partial dentures	23 (3.7)	20 (86.9)	
	Fixed partial dentures	13 (2.1)	3 (23.0)	

Variables were identified by chi-square difference testing; *n* = number of participants; no. of xerostomia cases = number of xerostomia cases; * *p*-values were significant (*p* < 0.05); ** *p*-values were significant (*p* < 0.001).

**Table 3:** The common risk factors ^a^ for oral function problems based on demographic and health behavior variables.

**Independent Factors**	**Number of Cases with Oral Function Problems**	**Speaking Problem**	**Tasting Problem**	**Swallowing** **Problem**	**Chewing Problem**
	No problems (%)	601 (96.5)	457 (73.4)	253 (40.6)	619 (99.4)
	Have problems (%)	22 (3.5)	166 (26.6)	370 (59.4)	4 (0.6)
	Categories	OR (95% CI)	OR (95% CI)	OR (95% CI)	OR (95% CI)
Xerostomia	No (reference)	3.31 * (1.11–9.80)	5.12 ** (3.26–8.06)	3.59 ** (2.32–5.53)	3.34 * (1.15–5.82)
	Yes				
Sex	Male (reference)	1.29 (0.45–3.69)	0.79 (0.52–1.20)	0.99 (0.65–1.51)	0.98 (0.35–2.74)
	Female				
Age (years)	50–59 (reference)	-	-	-	-
	60–69	1.69 (0.27–10.27)	0.81 (0.45–1.45)	0.95 (0.52–1.73)	1.50 (0.33–6.84)
	Over 69	2.54 (0.55–11.77)	0.71 (0.43–1.16)	1.00 (0.60–1.64)	1.34 (0.35–5.12)
Years since diabetes diagnosis	0–5 (reference)	-	-	-	-
	6–10	0.81 (0.17–3.67)	2.00 * (1.05–3.81)	1.69 (0.93–3.04)	1.84 (0.19–7.85)
	Over 10	1.69 (0.44–6.48)	2.69 * (1.43–5.07)	1.65 (0.91–2.98)	7.17 (0.91–8.12)
Denture wearing	No (reference)	0.98 (0.12–7.69)	1.77 (0.86–3.64)	1.54 (0.73–3.23)	5.45 * (1.65–7.93)
	Yes				
Type of denture	None (reference)	-	-	-	-
	Complete dentures	8.10 * (2.88–13.95)	4.99 (0.99–25.09)	2.00 (1.50–5.22)	25.90 ** (4.28–56.61)
	Removable partial dentures	2.00 (0.30–4.52)	2.18 (0.87–5.44)	2.62 * (1.08–6.37)	2.35 (0.29–19.06)
	Fixed partial dentures	1.00 (0.42–8.23)	0.41 (0.05–3.23)	0.89 (0.19–4.10)	4.31 (0.51–36.17)

^a^ The common risk factors were identified by bivariable logistic regression; OR = odds ratio; 95% CI = 95% confidence interval; * *p*-values were significant (*p* < 0.05); ** *p*-values were significant (*p* < 0.001).

**Table 4:** The common risk factors ^a^ for xerostomia based on demographic and health behavior variables.

Independent Factors	Categories (Total *n* = 623)	OR	95% CI	*p*-Value
Sex	Male (reference)	1.57	1.13–2.18	0.007 *
	Female			
Age (years)	50–59 (reference)	-	-	-
	60–69	2.10	1.36–3.22	0.001 *
	Over 69	2.55	1.95–3.53	0.005 *
Toothpaste (type 1)	SLS-free (reference)	1.00	0.59–1.68	0.082
	Containing SLS			
Toothpaste (type 2)	Spicy herbal extracts-free (ref.)	1.86	0.82–1.90	0.039 *
	Containing spicy herbal extracts			
Toothpaste (type 3)	Artificial sweeteners-free (ref.)	0.87	0.62–1.21	0.041 *
	Containing artificial sweeteners			
Education	None (reference)	-	-	-
	Elementary school	0.45	0.10–2.01	0.297
	High School	0.48	0.18–1.27	0.143
	Bachelor’s degree	0.62	0.24–1.57	0.314
	Higher than bachelor’s degree	0.50	0.19–1.30	0.158
Years since diabetes diagnosis	0–5 (reference)	-	-	-
	6–10	2.36	1.41–3.95	0.001 *
	Over 10	7.42	4.46–12.34	<0.001 **
HbA1c (%)	≤6.5 (reference)	-	-	-
	6.6–6.9	1.64	0.90–3.00	0.106
	≥7	4.96	3.02–8.13	<0.001 **
Having systemic diseases other than diabetes	No (reference)	6.25	3.60–10.86	<0.001 **
	Yes			
Systemic diseases other than diabetes	None (reference)	-	-	-
	Hypertension	5.45	3.58–8.28	<0.001 **
	Dyslipidemia	4.06	2.79–5.90	<0.001 **
	Cardiovascular disorders	13.26	1.64–26.73	0.015 *
	Thyroid disorders	2.70	1.10–6.62	0.030*
	Hematologic disorders	4.39	1.15–16.74	0.030 *
	Renal disorders	2.65	1.58–4.46	<0.001 **
	Respiratory disorders	1.37	0.79–2.40	0.259
	Allergy	1.27	0.83–1.93	0.262
	Gout	3.50	1.80–6.80	<0.001 **
Having medications	No (reference)	4.21	2.58–6.88	<0.001 **
	Yes			
Medications	None (reference)	-	-	-
	Antihypertensive medication	5.86	3.85–8.91	<0.001 **
	Antidyslipidemic agents	4.28	2.97–6.16	<0.001 **
	Antiplatlet and anticoagulant medication	2.35	1.68–3.30	<0.001 **
	Pain medication	1.02	0.72–1.44	0.907
	Gastrointestinal agents	1.25	0.46–3.42	0.654
	Cardiovascular medication	2.86	0.83–9.89	0.096
	Antihistamine	0.98	0.60–1.59	0.939
Smoking (cigarettes per day)	Never (reference)	16.49	13.80–17.48	<0.001 **
	1–5			
Alcohol consumption	No (reference)	6.73	3.99–11.35	<0.001 **
	Yes			
Alcohol consumption frequency	Never (reference)	-	-	-
	Monthly or less	6.54	3.13–13.65	<0.001 **
	2–4 times a month	5.15	2.32–11.44	<0.001 **
	2–3 times a week	21.10	2.68–26.18	0.004 *
	4 or more times a week	8.44	0.93–16.09	0.057
Denture wearing	No (reference)	3.94	2.00–7.74	<0.001 **
	Yes			
Type of denture	None (reference)	-	-	-
	Complete dentures	7.88	2.01–8.93	0.039 *
	Removable partial dentures	9.41	6.01–12.01	0.009 *
	Fixed partial dentures	2.22	1.78–6.62	0.001 *
Types of toothpastes	Subcategories	OR	95% CI	*p*-Value
Toothpaste containing SLS (type 1: *n* = 555)	HbA1c ≤ 6.5% (*n* = 122) (reference)	0.24	0.14–1.40	0.071
	HbA1c ≥ 6.6% (*n* = 433)			
Toothpaste containing spicy herbal extracts (type 2: *n* = 451)	HbA1c ≤ 6.5% (*n* = 97) (reference)	4.34	1.94–5.76	<0.001 **
	HbA1c ≥ 6.6% (*n* = 354)			
Toothpaste containing artificial sweeteners (type 3: *n* = 255)	HbA1c ≤ 6.5% (*n* = 55) (reference)	0.36	0.17–0.73	0.005 *
	HbA1c ≥ 6.6% (*n* = 200)			

^a^ The common risk factors were identified by bivariable logistic regression; OR = odds ratio; 95% CI = 95% confidence interval; * *p*-values were significant (*p* < 0.05); ** *p*-values were significant (*p* < 0.001).

**Table 5:** Prognostic risk factors ^a^ for xerostomia based on demographic and health behavior variables.

Independent Factors	Categories	OR	95% CI	*p*-Value
Sex	Male (reference)	1.36	1.21–2.61	<0.001 **
	Female			
Age (years)	50–59 (reference)	-	-	-
	60–69	1.51	1.29–1.89	0.019 *
	Over 69	2.31	1.84–2.53	0.026 *
Toothpaste (type 1)	SLS-free (reference)	1.40	0.13–1.67	0.096
	Containing SLS			
Toothpaste (type 2)	Spicy herbal extracts-free (ref.)	9.32	3.46–15.25	0.032 *
	Containing spicy herbal extracts			
Toothpaste (type 3)	Artificial sweeteners-free (ref.)	0.35	0.02–4.82	0.013 *
	Containing artificial sweeteners			
Years since diabetes diagnosis	0–5 (reference)	-	-	-
	6–10	1.08	0.03–1.18	<0.001 **
	Over 10	2.40	0.23–4.68	0.001 *
HbA1c (%)	≤ 6.5 (reference)	-	-	-
	6.6–6.9	5.10	1.05–9.21	<0.001 **
	≥ 7	8.17	2.08–12.34	<0.001 **
Type of denture	None (reference)	-	-	-
	Complete dentures	3.66	1.51–5.96	0.003 *
	Removable partial dentures	8.59	1.60–12.55	0.001 *
	Fixed partial dentures	1.97	1.00–19.95	0.004 *

^a^ Prognostic risk factors were identified by multiple logistic regression analysis (estimates from multivariate logistic regression analysis including terms for education, systemic diseases other than diabetes, medications, smoking, and alcohol consumption); OR = odds ratio; 95% CI = 95% confidence interval; * *p*-values were significant (*p* < 0.05); ** *p*-values were significant (*p* < 0.001).

## Data Availability

All data used and/or analyzed during the current study are available from the corresponding authors upon reasonable request.
